# Provincial prenatal record revision: a multiple case study of evidence-based decision-making at the population-policy level

**DOI:** 10.1186/1472-6963-8-266

**Published:** 2008-12-19

**Authors:** Nancy Edwards, Sonia Semenic, Shahirose Premji, Phyllis Montgomery, Beverly Williams, Joanne Olson, Omaima Mansi

**Affiliations:** 1 University of Ottawa, 451 Smyth Road, Roger Guindon Pavilion 1118, Ottawa, Ontario, K1H 8M5, Canada; 2 School of Nursing, McGill University, 3506 University Street, Montreal, Quebec, H3A 2A7, Canada; 3 Faculty of Nursing, University of Calgary, Health Region, Calgary, 2500 University Drive NW, Calgary, Alberta, T2N 1N4, Canada; 4 School of Nursing, Laurentian University, Ramsey Lake Road, Sudbury, Ontario, P3E 2C6, Canada; 5 Faculty of Nursing, University of Alberta, 114th Street, 3rd Floor Clinical Sciences Building, Edmonton, Alberta, T6G 2G3, Canada

## Abstract

**Background:**

There is a significant gap in the knowledge translation literature related to *how *research evidence actually contributes to health care decision-making. Decisions around what care to provide at the population (rather than individual) level are particularly complex, involving considerations such as feasibility, cost, and population needs in addition to scientific evidence. One example of decision-making at this "population-policy" level involves what screening questions and intervention guides to include on standardized provincial prenatal records. As mandatory medical reporting forms, prenatal records are potentially powerful vehicles for promoting population-wide evidence-based care. However, the extent to which Canadian prenatal records reflect best-practice recommendations for the assessment of well-known risk factors such as maternal smoking and alcohol consumption varies markedly across Canadian provinces and territories. The goal of this study is to better understand the interaction of contextual factors and research evidence on decision-making at the population-policy level, by examining the processes by which provincial prenatal records are reviewed and revised.

**Methods:**

Guided by Dobrow et al.'s (2004) conceptual model for context-based evidence-based decision-making, this study will use a multiple case study design with embedded units of analysis to examine contextual factors influencing the prenatal record revision process in different Canadian provinces and territories. Data will be collected using multiple methods to construct detailed case descriptions for each province/territory. Using qualitative data analysis techniques, decision-making processes involving prenatal record content specifically related to maternal smoking and alcohol use will be compared both within and across each case, to identify key contextual factors influencing the uptake and application of research evidence by prenatal record review committees. All study participants will be required to give written informed consent prior to participating in data collection.

**Conclusion:**

This study will advance knowledge in the field of evidence-based decision-making by illustrating the complex interaction of contextual factors and evidence on health policy decision-making by provincial-level committees. By increasing the transparency of decision-making within provincial prenatal record committees, this study will help inform more effective strategies for enhancing the integration of best-practice evidence into prenatal records.

## Background

Despite increasing emphasis on the production of high-quality research evidence in health care, much is unknown about *how *evidence is actually used in decision-making [1]. Decisions around what care to provide at the population (rather than individual) level are particularly complex, involving considerations such as feasibility, cost, population needs and political attractiveness in addition to scientific evidence [2-4]. As mandatory medical reporting forms, provincial prenatal records are potentially powerful vehicles for promoting best-practice recommendations. Decisions made by provincial-level review committees about what screening questions and intervention prompts to include on prenatal records are vital, as these documents guide routine prenatal care and have population-wide reach. The extent to which current prenatal records reflect the evidence base around important risk factors such as maternal smoking and alcohol use varies markedly across Canadian provinces and territories [5], begging the question of how research evidence penetrates the prenatal record revision process. Since the available research base for prenatal risk factors is presumably the same for all prenatal record review committees, marked differences in the content of provincial prenatal records point to differences in the broader decision-making environment within which these forms are developed and updated. Decision-making related to provincial prenatal record content is particularly pertinent as pregnancy and childbirth remain the most common reasons for hospitalization among Canadian women [6]; these mandatory forms are widely used by health care providers across a variety of disciplines; however the processes by which research evidence is integrated into such provincial-level health policy decisions remains poorly understood [7].

### Aims

To gain an in-depth understanding of the influence of contextual factors on evidence use by provincial/territorial prenatal record review committees, this study will focus on two specific exemplars: the uptake of research evidence related to maternal smoking, and alcohol consumption during pregnancy. The scientific evidence identifying smoking and alcohol use as risk factors for adverse obstetrical outcomes is well-documented and long-standing [8,9] meriting closer examination of how and why prenatal screening for these two factors varies among provinces. Specific objectives of this study are to:

1) Describe and compare how provincial prenatal records are reviewed and updated across selected Canadian provinces;

2) Identify specific contextual factors in the decision-making environment that impede or facilitate the use of research evidence during the prenatal record revision process, as well as alternative sources of evidence contributing to decision-making within provincial prenatal record committees; and

3) Explore the relative impact of internal versus external contextual factors on the integration of research evidence in provincial practice policies such as the prenatal record.

Four main research questions underlie these study objectives:

What are the processes and who are the actors involved in developing and revising provincial prenatal records?

How are decisions made about the content of provincial prenatal records related specifically to maternal smoking and prenatal alcohol?

What key contextual factors influence the uptake of research evidence during the prenatal record revision process?

What other factors in the decision-making environment at the provincial level may account for variations in the evidence base of prenatal records across provinces?

### Summary of the Literature

#### Canadian provincial prenatal records

Standardized provincial prenatal assessment forms are universally used in most provinces and territories in Canada to serve as a written health record which may be transferred from one part of the health care system to another (e.g., obstetrician's office to hospital obstetrical unit) and provide data for routine surveillance of perinatal risk factors. The government prenatal records for each province and territory typically include a comprehensive health history form completed by health care providers at the first prenatal visit, as well as additional forms for documentation of on-going prenatal care at each subsequent encounter. By providing health care practitioners with reminders and prompts, provincial prenatal records outline the content of routine prenatal care and play a key role in guiding health professionals in what to assess and how to intervene with pregnant women. Physician reminders and prompts have been identified as one of the most effective mechanisms for transferring evidence into routine practice in preventative care [10]. As standardized assessment tools that are part of regulated surveillance systems, provincial prenatal records are potentially influential conduits for population-wide dissemination of best-practice recommendations [11]. Each province and territory in Canada has its own unique prenatal record, except for Yukon Territory (which uses British Columbia's prenatal record forms); and New Brunswick (where prenatal records are developed at the regional level). Decisions about what to include and what to exclude in the way of risk factors and prompts on the prenatal record forms are typically made by a provincially-mandated multidisciplinary committee [5]. Canadian provinces and territories vary in the frequency that they review and update their prenatal records, but in general these provincially-regulated forms are formally revised and reprinted every few years.

Despite the potential for manipulating prenatal records to mandate evidence-based clinical care, literature examining and comparing the content and quality of Canadian prenatal records is virtually non-existent. A recent examination of Canadian prenatal records for their content related to maternal smoking found marked variation in the application of available research evidence across provinces [5]. In the published literature, one study was found that compared national prenatal care clinical practice guidelines from four countries including Canada, although the Canadian documents surveyed (Healthy Beginnings: Guidelines for Care during Pregnancy and Childbirth [12] and The Canadian Guide to Clinical Preventative Health Care [13]) do not necessarily model the content for the provincial and territorial prenatal records. This comparison found little consistency either within or across countries in terms of their recommendations for routine prenatal care, and called for re-examination of the evidence-base of the national prenatal care guidelines [14]. Similarly, a survey of routine prenatal care guidelines in Australia found local protocols varied widely across institutions and were not consistent with national policies or research evidence. This study highlighted the need to develop standardized prenatal care recommendations based on systematic reviews of the evidence [15].

#### Evidence-based decision-making

A central focus of the study of knowledge utilization in health care is to better understand why available research evidence is not readily adopted. Few studies, however, have explored precisely how research evidence is evaluated and integrated into provincial-level practice policies. This specific area of inquiry is uniquely situated between two more extensively-researched bodies of literature in the field of evidence-based decision-making: 1) the development and implementation of clinical practice guidelines (CPGs), and 2) evidence-based health policymaking. The following text addresses each of these topics in further detail.

Evidence-based CPGs are increasingly viewed as important decision-making tools in the quest to improve the quality and effectiveness of health care [16-18]. Growing attention is being paid to the actual processes involved in developing and maintaining the content of CPG's [19]. For example, an international survey of 18 national-level guideline development programs (including Canada's Cancer Care Ontario Practice Guidelines Initiative) found most guideline development groups consisted of 10–20 members from three to five disciplines, and offered training in guideline development methodology to group members. In addition, most of these groups based their recommendations on systematic reviews of the research evidence as well as formal or informal consensus procedures when evidence was controversial or lacking [20]. Inconsistencies in CPG recommendations on the same topic from different countries, however, are common, and have been attributed to such factors as differing interpretations of the research evidence, unsystematic guideline development methods, and cultural differences between health care systems [21]. In addition to the scientific evidence, the guideline development process typically considers other sources of evidence such as clinical experience, patient preferences, resource implications and feasibility [18,22]. The extent to which CPGs incorporate research evidence also may vary according to such issues as the perceived purpose of the guidelines, the resources available to develop the guidelines, and the approach used to generate group consensus on recommendations [23]. The use of formal consensus-building strategies (e.g., the nominal group technique) is considered integral to the quality of practice guidelines such as CPG's, consensus statements and health technology assessments [18]. Much remains to be known about the intricate decision-making processes involved in the development of such clinical practice policies. An investigation of the impact of small group processes on the development of CPGs by multidisciplinary teams revealed the influence of clear status hierarchies, with higher-status professionals (e.g., medical experts) contributing more to group discussions and decision-making than nurses or general practitioners [24,25]. Another recent ethnographic study of multi-disciplinary guideline development groups [26] observed the entire development process of two different CPGs, and identified distinct "repertoires of evaluation" used by group members when developing their recommendations (e.g., robustness of the evidence, practicality in routine patient care, political acceptability of the evidence). Moreira [26] reported that group members altered their participation in the decision-making process according to their comfort with the particular evaluation mode predominant in the group discussion, and that the resulting practice recommendations represented combined knowledge from the different "worlds" of science, practice, politics and process.

The important question of when and how CPGs should be modified and updated following their development and dissemination has received considerably less attention, although such formal practice guidelines are expected to be as evidence-based as feasible [19,27]. The international survey of national guideline development programs cited above found half did not have formal update procedures [20]. An examination conducted in 2001 of the validity of all 17 CPGs developed by the US Agency for Healthcare Research and Quality found almost half the guidelines were obsolete within 5.8 years, leading the authors to recommend that guidelines be reassessed every three years as a general rule [28]. Although regularly-scheduled literature updates have been recommended to maintain CPG validity and minimize the burden of rigorous systematic re-review [19,29], the resources and motivations to do so may vary across different types of guideline development groups (e.g., local vs. national).

Whereas standardized provincial prenatal records serve in essence as clinical practice guidelines, their mandatory use as part of the patient record and additional role in health surveillance adds complexity to decision-making around their content. However findings from the field of CPG development and maintenance suggest that various issues may influence decision-making around provincial prenatal content, such as the composition and dynamics of the committee responsible for reviewing and updating the prenatal records; the different sources and types of evidence considered in the decision-making process; and situational factors such as time and resources allocated to the review process.

The second topic, evidence-based health policymaking, has been implicated in decision-making in health care. Growing demand for a return from investments in health services research has increased the need for transparency and rationalization of health policy decision-making [4]. The term *health policy *is used variably in the evidence-based decision-making literature to refer to broad political actions taken by governments and other decision makers to improve the health of populations (e.g., regionalization of care), as well as more specific clinical practice directives aimed at the population level (e.g., screening recommendations for breast cancer or other diseases). Our study focus falls more closely within the latter definition, which views evidence-based *health policy *(in contrast to evidence-based *clinical care*) as the application of best current knowledge to the health needs and values of populations rather than individual patients [3].

To date, studies in the area of evidence-based health care policymaking have focused largely on increasing research uptake in the policy setting by means of strengthening relationships between policy makers and researchers [30]. Few studies have directly examined how and under what conditions research evidence is actually used to shape health policy decision-making. Dobbins et al. [31] found the strongest predictor of whether provincial public health decision-makers used systematic reviews to influence their policy decisions was perceived organizational value for the use of research evidence, and identified the need to better understand how the organizational context within public health units impacts on individual policy decision-makers. A study of the use of evidence in the development of local "health improvement programs" in the UK found that decision-makers relied on a mix of experiential (i.e., based on professional opinion and tacit knowledge) and empirical (i.e., based on published research or guidelines) evidence, and that national CPGs were a particularly influential form of evidence in these public health policy settings [1]. However this study also revealed that public health decision makers considered improving health, reducing health inequalities and encouraging partnerships in the provision of care to be more important goals than efficient provision of health services, thus underscoring the role of political values in shaping health policy decisions [1]. Black [32] also noted that health care policymakers often have goals other than maximizing clinical effectiveness, supporting the need to further examine competing pressures within the decision-making context. In contrast to individual patient care, health care policies aimed at the population level must address broader issues such as universal access, value for money, and the needs/values of the population [3,4]. Practice recommendations that define clinical care for entire populations, such as provincial prenatal records, thus present a unique case study for evidence-based decision-making. Dobrow et al. [2] used the example of policy development for colorectal cancer screening to demonstrate how the decision-making context differs for evidence-based decision-making at the individual clinician level, as compared to health policy decision-making that targets entire populations. Health care decisions at this "population-policy level" affect more people, require more explicit justification, and are influenced by a myriad of competing issues (e.g., strength of the evidence, feasibility of implementation, political attractiveness) [2,31]. Dobrow et al. defined "context" as all factors within an environment where a decision is made and further distinguished between a modifiable "internal" context within which the decision-making process occurs and a broader, fixed, "external" decision-making context influencing health policy decision outcomes. Criteria for universal screening for a particular disease or risk factor also include the availability of a valid screening test, acceptability of routine screening to patients and health care providers, evidence that the benefits of routine screening outweigh potential harms, and demonstrated cost-effectiveness [33,34], adding to the variety of evidence that may be weighed to justify health policy decisions.

Findings from the field of evidence-based health policy point to the decision-making environment at the provincial level as an important influence on what types and sources of evidence are considered in decisions around prenatal record content. An important gap that remains in the literature is the relative influence of different types or levels of contextual factors on the uptake and application of evidence in health policy decision-making. By closely examining how provincial prenatal records are reviewed and revised, this study aims to identify key contextual influences on evidence-based decision-making that can help inform strategies to increase research uptake at the population-policy level. The development of standardized provincial prenatal records offers an intriguing example of health policy decision-making, particularly as the forms function as both national practice guidelines and as a mandatory clinical documentation system. Prenatal records also address a broad scope of clinical parameters, allowing comparison of evidence integration across different content areas. To more closely examine decision-making processes within provincial prenatal record review committees, this study will focus specifically on the uptake and application of research evidence related to the prenatal assessment of maternal smoking and alcohol use.

#### Prenatal assessment of maternal smoking and alcohol use

The Canadian Guide to Clinical Preventive Health Care advocates routine prenatal screening and counseling for the behavioral risk factors of both maternal smoking and alcohol use [13]. According to the Public Health Agency of Canada's 2005 Report on Maternal Child Health in Canada, maternal smoking and alcohol consumption rates have been decreasing but remain a public health concern [35]. In 2003, 14% of recent mothers reported smoking daily during pregnancy and about 14% also reported drinking alcohol when pregnant [36].

Maternal smoking remains an important modifiable risk factor for adverse perinatal outcomes as well as for as longer-term mother and child health problems [37-39]. Health care costs attributable to smoking during pregnancy and postpartum are substantial, with low infant birth weight carrying the highest economic burden [40,41]. The implementation of clinical systems to systematically assess and document patient smoking status has been shown to significantly increase the rate at which clinicians intervene with patients who smoke [42]. Moreover, pregnancy is well-recognized as an opportune "teachable moment" for smoking cessation due to enhanced maternal motivation to quit and the strong social stigma associated with smoking when pregnant [43,44]. Best-practice guidelines for perinatal smoking cessation and relapse prevention interventions based on systematic reviews of the research evidence are widely available in the literature (e.g., [45-49]), and Canada boasts numerous national and provincial programs for promoting smoking cessation during pregnancy as a result of federal tobacco control initiatives in the early 1990's [50]. Recommended strategies to reduce maternal smoking derived from this body of research include screening for tobacco use at each prenatal visit and using multiple response formats to enhance disclosure; assessing pre-pregnancy smoking history in addition to exposure to second-hand tobacco smoke; and offering tobacco cessation interventions to smoking women as well their smoking partners.

Despite the availability of research evidence related to effective screening and interventions to reduce maternal smoking, current Canadian prenatal records vary markedly in the extent to which they incorporate these best-practice recommendations. A recent comparison of provincial and territorial prenatal records from all Canadian provinces and territories found that all the prenatal record forms included at least one question assessing maternal tobacco use at the first prenatal visit, but only two of the forms provided prompts for on-going monitoring of maternal smoking throughout the rest of pregnancy and only two included a prompt to refer smokers for specialized smoking cessation support [5]. Several of these prenatal records had been revised within the past few years, suggesting that available research evidence related to maternal smoking had somehow failed to penetrate the prenatal record revision process.

The other behavioral risk factor of this study's interest is maternal alcohol use. Alcohol use during pregnancy is a significant and preventable public health issue that can result in a lifelong burden of psychological, emotional, and financial costs to the affected individuals and families [51-53]. Prenatal exposure to alcohol is associated with a continuum of effects including growth deficits, dysmorphology, and complex patterns of behavioral and cognitive difficulties resulting from central nervous system damage during fetal life [54]. High blood-alcohol concentration is the most significant risk factor for fetal alcohol spectrum disorders (FASD) and is related to timing of exposure during fetal development, the pattern of consumption of alcohol (e.g., binge drinking, that is four or more drinks per occasion), and frequency of alcohol use [55]. Guidelines developed by a subcommittee of the Public Health Agency of Canada's National Advisory Committee on FASD recommends that all pregnant and post-partum women be screened for alcohol use [55]. A comprehensive assessment of alcohol use during pregnancy is advocated to identify high risk drinking patterns, discuss prenatal alcohol exposure and offer effective counseling to decrease alcohol intake [56-58]. Of numerous screening questionnaires that have been developed to assess alcohol use, the brief, four-question T-ACE scale has been determined as most accurate in detecting current alcohol consumption, heavy alcohol use and risk drinking among pregnant women [59]. A preliminary review of maternal alcohol use questions on current Canadian prenatal records revealed that similar to the risk factor of maternal smoking, provincial prenatal records vary considerably in their screening questions and intervention prompts related to maternal alcohol consumption. For example, whereas four provinces/territories examined include the T-ACE score in their prenatal record forms, several others assess current maternal drinking status with a simple yes/no question. In reviewing all of the provincial and territorial forms, we noted some interesting parallels in the evidence-based content of smoking and alcohol screening questions. With a few exceptions, those provinces with weak questioning related to maternal smoking were similarly weak regarding screening for alcohol use. This provides another indication that contextual factors at the provincial level are influencing the use of evidence in this example of population-policy decision-making.

### Conceptual Framework

**Figure 1:**
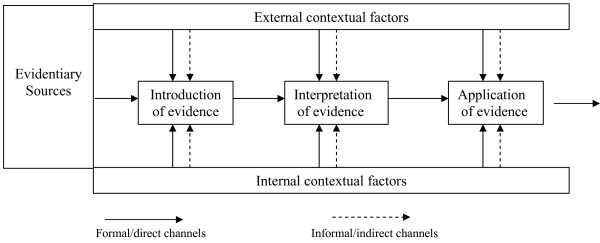
**Conceptual framework for context-based evidence-based decision-making**. Reprinted from *Social Science and Medicine*, vol. 58, Dobrow M, Goel V, Upshur REG. Evidence-based health policy: Context and utilization, pp.207–17, copyright 2004, with permission from Elsevier.

This study of evidence-based decision-making by provincial prenatal record review committees will be guided by Dobrow et al.'s (2004) conceptual framework for "context-based evidence-based decision-making" [2] (Figure 1). Dobrow et al. illustrated their model using an example of health care decision-making at the "population-policy" level (the development of a colorectal cancer screening policy). According to this process-oriented model, both internal and external contextual factors impact on what individual sources of evidence are collectively weighed and prioritized to justify decisions. The *internal decision-making context *refers to the environment in which decisions are *made *and includes the purpose of the decision-making activity, participants' roles in the decision-making process, and the strategies used to arrive at the decision outcome (i.e., the specific questions around maternal smoking and alcohol use to be included on the provincial prenatal records). The individual participants on the prenatal record revision committees may influence the decision-making process through personal characteristics or relationships they bring to the table, as demonstrated in the previously-mentioned studies [23,24] of how small group processes influence CPG development. The *external decision-making context *refers to the provincial environment in which the decisions are *applied*, and involves more fixed, uncontrollable factors such as patterns of service delivery and dynamics among health professional organizations; as well as economic, regulatory and other sociopolitical features that may influence policy alternatives. Dobrow et al.'s model traces how internal and external contextual factors impact three main stages of evidence utilization: 1) *introduction *of evidence, that is, how evidence is identified and brought to the decision-making table; 2) *interpretation *of evidence, which refers to how the internal and external validity of the evidence is evaluated; and 3) *application *of evidence (i.e., the ultimate influence each individual source of evidence has on the decision outcome).

This framework for evidence-based decision-making is particularly useful for understanding why best-practice recommendations related to maternal smoking and alcohol consumption may have been differentially applied by provincial prenatal record review committees, as it explicitly moves the focus of inquiry beyond "the evidence" to specific contextual features within the broader policy environment. Dobrow et al.'s model suggests numerous propositions that may explain why some prenatal records have more detailed and evidence-based questioning of risk factors than others. For example, political pressure from provincial medical associations to minimize government control over medical practice (i.e., external decision-making context) may act to decrease the number and specificity of prenatal record assessment questions and prompts. Conversely, provinces with more time and money allocated to the prenatal record revision process and/or greater capacity of committee members to assess the available research evidence (i.e., internal decision-making context) would be expected to have more evidenced-base prenatal records. An important question to address is how factors from both these different decision-making contexts are collectively weighed to justify health policy decisions. Because we are examining the integration of two areas of research evidence (maternal smoking and alcohol use) in the prenatal record forms, our methods will allow us to decipher the relative influence of the internal and external decision-making contexts both within and across provincial prenatal record review committees, helping to identify key contextual factors influencing decision-making at the population-policy level.

## Methods

### Design

A multiple case study with embedded units of analysis will be used to examine and compare decision-making processes within and across provincial prenatal record review committees. The case study approach using mixed-methods is most valuable when the questions being posed require investigation of a real-life event in detail; where the focus is on "how" and "why" questions; when boundaries between phenomena and their context are not clear; and when the investigators have little control over events [60,61]. The diverse group of organizations and individuals that work together to review and revise prenatal records within each Canadian province/territory offer ideal natural "laboratories" for detailed analyses of population-policy decision-making. Dobrow et al. [2] noted the conceptual difficulty in distinguishing evidence from context, and thus the case study design helps tease apart their joint influence on decision-making. The multiple (or "comparative") case study seeks both *literal *replication of findings (obtaining the same results across similar cases) and *theoretical *replication (obtaining different results but for predictable reasons), allowing exploration of the study's theoretical underpinnings [60]. Study cases are carefully selected to pursue different patterns of theoretical replication; a larger number of cases (i.e., 6–10) provide more compelling support for predicted findings. Guided by the framework for context-based evidence-based decision-making, the overarching question for our study is, "What is the influence of contextual factors on the uptake and application of evidence by provincially-mandated prenatal record review committees?" The main unit of analysis for this case study is the prenatal record revision process within each province/territory. To be able to examine intricate decision-making processes in depth, two sub-units of analysis (decisions around prenatal record content pertaining to both maternal smoking and alcohol use) will be embedded within each case study. Ethical approval for this study has been granted by the University of Ottawa Research Ethics Board and the affiliated research ethics boards from institutions of all the study's co-principal and co-investigators [see Additional file 1, Additional file 2, Additional file 3, Additional file 4 and Additional file 5].

### Setting and Sample

We purposefully selected six Canadian provinces that varied in the extent to which their prenatal record content related to maternal smoking and alcohol use was evidence-based, as reflected by questions and intervention prompts included on current prenatal records from each province and territory in Canada. We considered the extent to which the prenatal forms integrated available research evidence related to maternal smoking questions as "weak" if the prenatal record simply inquired about current smoking status, and "strong" if there were additional questions addressing issues such as previous smoking history and exposure to environmental tobacco smoke, and if the prenatal record provided for ongoing monitoring and/or prompted referral of smokers to specialized smoking cessation resources. Similarly, we considered the extent to which prenatal record content related to alcohol consumption was evidence-based to be "weak" if questions simply inquired about present alcohol use, and "strong" if the T-ACE scale also was included as part of routine prenatal assessment. Of the six provinces chosen for comparison, two had prenatal records with strong screening questions for both smoking and alcohol use; two had weak questioning related to both smoking and alcohol use; one had strong smoking questions but weaker alcohol-related questions; and one had weak assessment of maternal smoking but stronger questions related to alcohol use. The study's embedded design thus allowed for the examination of 12 separate decision-making events *across *these six provinces, in addition to comparing contextual influences on decision-making around maternal smoking vs. alcohol use questions and prompts *within *each of the case provinces. Other important dimensions of comparison across the selected case provinces may come to light as our case study unfolds, such as size of the province and type of organizational body responsible for maintaining the prenatal records.

### Data Collection

Multiple sources of data will be used to explore internal and external contextual factors at play during the prenatal record revision process [60]. The principle sources of evidence for this study will be in-depth interviews, survey questionnaires, and document review. A case study protocol will be developed to ensure systematic and comparable collection of data across different cases [60]. Preliminary telephone interviews will be conducted with identified contacts within each of the selected case provinces to obtain a basic profile of the province's prenatal record review system, and to identify potential key informants for in-depth interviews. A combination of purposive and snowballing sampling will be used to obtain a sample of key informants from each of the case study sites. Informants may include (but are not limited to) the chairperson as well as members of the provincial prenatal record revision committee, clinical experts consulted by the committee for the specific topics of maternal smoking or alcohol use, policy makers in government responsible for provincial maternal child health policies, and health professional associations/colleges responsible for regulatory standards. Key informants will be interviewed in their language of choice (English or French) either by telephone or face-to-face during site visits to each case province, using a semi-structured interview guide. Interviews will address potential internal and external contextual factors as well as different types and sources of evidence that may have contributed to decisions around prenatal record content made during the province's most recent prenatal record revision. These interviews will take approximately one hour to complete. We anticipate interviewing an average of 12 key informants per case province. All interviews will be audio-taped and transcribed verbatim. Following the semi-structured interviews, participants will be asked to complete demographic information and a survey questionnaire further exploring their perceptions of how decisions around prenatal record content related to maternal smoking and alcohol use were made. This theoretically and empirically-based questionnaire will be reviewed by an expert panel for face and content validity, refined, and then piloted within two provinces not included as study sites where the investigators have personal contacts with prenatal record review committee members.

Pertinent documents (e.g., meeting agendas and minutes, policy briefs, drafts of prenatal record revisions, published studies or provincial guidelines used as reference materials, lists of provincial committee members) will be examined (given organizational permission) during a site visit to the provincial organizations responsible for the prenatal record review process in each of the six case provinces. When permitted, documents and records will be copied to include as part of the case study data base. The purpose of the site visits are to interview key informants, collect additional corroborating documentary data, gain a first-hand sense of the myriad actors and organizations involved in the prenatal record review process, and seek potential opportunities to directly observe decision-making processes in provinces currently engaged in prenatal record review. Due to the large volume of data to be collected and the variety of organizational settings that may need to be visited per province, each site visit will be conducted by the research associate accompanied by at least one of the study investigators. Each site visit will be scheduled over two days. Those conducting each site visit will be asked to prepare a brief report including their field notes recording their impressions and observations of the visit. This report will be shared with provincial prenatal record committee members to verify that we have provided an accurate record of decision-making processes. The research associate will be responsible for organizing a case study database for each case province, and for maintaining additional field diaries throughout the study to record observations and track events that may be of relevance to findings and analysis.

### Data Analysis

Transcription of interviews and data analysis will proceed concurrently to permit follow-up of issues that might emerge from the data. Data will be transcribed and analyzed in their source language (i.e., the language in which the interviews were conducted). The content of interview transcripts, documents and observations will first be analyzed descriptively to characterize the prenatal record review process for each case province, and to develop a detailed narrative of how and why decisions around prenatal record content related specifically to maternal smoking and alcohol use were arrived at. The data will then be examined using a number of qualitative data analysis techniques and the analytic categories suggested by Dobrow et al.'s conceptual model, while also building on new insights that may arise from close familiarity with the data. For example, content analysis of the data will be conducted to identify different evidentiary sources weighed in the decision-making process, as well as contextual features of the decision-making environments. Contextual factors will be coded and categorized according to their type (i.e., internal vs. external decision-making context) as well as point of impact on individual sources of evidence (i.e., introduction, interpretation, or application of the evidence). New or more refined categories may be developed as needed. Data will be ordered and displayed using category matrices, graphics and frequency tables [62]. The analysis will be validated with key informants from the participating provinces, to help generate a rich understanding of decision-making related to prenatal record content within each case province. In the next phase, pattern-matching [60] will be used to compare and contrast decision-making processes both within each case province (i.e., for maternal vs. alcohol prenatal record content) and across the different case provinces. The mix of different contextual factors as well as their effects on evidence utilization will be tabulated using cross-site displays, to help generate inferences about the relative impact of the decision-making environment on the prenatal record revision process. Descriptive statistics will be used to analyze the questionnaire results, and case sets will be compared using non-parametric tests.

The four tests commonly used to ensure rigor in case study designs are construct validity, internal reliability, external validity and reliability [60]. Construct validity will be pursued by using multiple sources of evidence, establishing a chain of evidence, and having key informants review the draft case study report for their province. Internal validity will be addressed through pattern matching and seeking rival explanations. Word tables will be generated independently by investigators and where there are discrepancies, discussions will ensue to reach consensus. External validity will be strengthened through the use of multiple cases to pursue replication of predicted findings, and reliability will be pursued by developing and following a comprehensive case study protocol, maintaining detailed field notes and organizing a case study database [60].

## Discussion

This study addresses an important gap in the field of evidence-based decision-making by seeking to illustrate the complex influence of contextual factors on decision-making processes within provincial-level committees. It will build on Dobrow et al.'s framework, generating knowledge about specific contextual factors that maximize the uptake of evidence in population-policy decision-making. Specifically, this study will increase the transparency of decision-making within provincial prenatal record committees and will thus help to identify effective strategies for promoting the integration of research evidence into the mandatory and widely used prenatal records. More broadly, study findings will contribute to a model of evidence-based decision-making that can be adapted to the broader contextual features of policy environments, helping inform avenues for promoting research utilization for other types of committees developing provincial tools and programs. Examples include the development of mandatory core program guidelines in Ontario (legislation that requires health departments to provide stipulated programs and activities); and the development of surveillance tools for use across clinical practice settings such as injury surveillance, and patient safety monitoring tools.

## Conclusion

This proposed research directly meets the Canadian Institutes of Health Research's knowledge translation strategic direction of advancing research in the use of health-related knowledge across the various levels of decision-making in the health system. Therefore, the study results should advance our understanding of internal and external contextual factors that influence population-policy decision-making. As such, results are expected to be of interest to scientists working in the fields of knowledge translation, public and population health, and health services research.

## Abbreviations

CPGs: Clinical Practice Guidelines; FASD: Fetal Alcohol Spectrum Disorders

## Competing interests

The authors declare that they have no competing interests.

## Authors' contributions

All authors contributed to the conception and design of the study protocol. SS and NE drafted the protocol, with further input from all other authors. This manuscript was prepared by PM and SS. All authors read and approved the final manuscript.

## Pre-publication history

The pre-publication history for this paper can be accessed here:



## Supplementary Material

Additional file 1 **Ethics approval Ottawa additional file 1.**  Ethics approval to study protocol from the University of Ottawa research ethics board Click here for file

Additional file 2 **Ethics approval Laurentian additional file 2.**  Ethics approval to study from University of Laurentian research ethics board Click here for file

Additional file 3 **Ethics approval McGill additional file 3.**  Ethics approval to study protocol from McGill University research ethics board Click here for file

Additional file 4 **Ethics approval Alberta additional file 4.** Ethics approval to study protocol from University of Alberta research ethics board Click here for file

Additional file 5 **Ethics approval Calgary additional file 5.**  Ethics approval to study protocol from University of Calgary research ethics board Click here for file
