# Supplementary material for: Provincial prenatal record revision: a multiple case study of evidence-based decision-making at the population-policy level
Source: BMC Health Serv Res. 2008 Dec 19;8:266. doi: 10.1186/1472-6963-8-266 (PMC2642799; doi:10.1186/1472-6963-8-266)
Supplement: Additional file 1 — Ethics approval Ottawa additional file 1. Ethics approval to study protocol from the University of Ottawa research ethics board [file 1472-6963-8-266-S1.pdf]

April 7, 2008

Nancy Edwards
School of Nursing
Faculty of Health Sciences
University of Ottawa
451 Smyth Road, room 1118
Ottawa, On K1H 8M5

Shahirose Premji
School of Nursing
University of Calgary
2500 University Drive
Calgary

Object: Provincial Prenatal Record revision: A Multiple Case Study of Evidence-Based Decision-Making at the Population-Policy Level (File H 09-06-06)

Dear Pr. Edwards and Dr. Premji,

You will find enclosed the Health Sciences and Science Research Ethics Board renewal certification for your research project above-mentioned.

During the course of the study, any modifications to the protocol or forms may not be initiated without prior written approval from the REB. You must also promptly report to the REB all adverse events or experiences encountered by participants.

The renewal certification is retroactive to February 23, 2008 and valid until February 23, 2009. Please submit an annual status report to the Protocol Officer in February, 2009 to either close the file or request a renewal of ethics approval. This document can be found at: http://www.rges.uottawa.ca/ethics/application_dwn.asp

A copy of this renewal approval will be sent to Research Services, if necessary.

Please do not hesitate to contact me at extension 5387 if you should have any questions.

Sincerely,

Germain Zongo
Protocol Officer for Ethics in Research
For Dr. Daniel Lagarec, Chair of the Health
Sciences and Science REB

Université d'Ottawa University of Ottawa
Service de subventions de recherche et déontologie Research Grants and Ethics Services

HEALTH SCIENCES AND SCIENCE RESEARCH ETHICS BOARD

CERTIFICATION OF ETHICS APPROVAL

This is to certify that the University of Ottawa Health Sciences and Science Research Ethics Board (REB) examined the application for extension of ethics approval for the research project **Provincial Prenatal Record Revision: A Multiple Case Study of Evidence-Based Decision-Making at the Population-Policy Level (file H 09-06-06)** submitted by Pr. Nancy Edwards of the School of Nursing and Pr. Shahirose Premji of the University of Calgary. Sonia Semenic, Joanne Olson, Beverly Williams, Phyllis Montgomery and Omaima Mansi are co-investigators.

This project received initial ethics approval on March 23, 2007 by the REB as meeting appropriate ethical standards set out in the Tri-Council Policy Statement and in the Procedures of the University of Ottawa Research Ethics Boards. The University of Ottawa REB members accordingly gave it a one-year extension of ethics approval. This ethics renewal certification is retroactive to March 23, 2008 and valid until March 23, 2009.

Germain Zongo
Protocol Officer for Ethics in Research
For Dr. Daniel Lagarec, Chair of the
Health Sciences and Science REB

April 7, 2008
Date

550, rue Cumberland 550 Cumberland Street
Ottawa (Ontario) K1N 6N5 Canada Ottawa, Ontario K1N 6N5 Canada

(613) 562-5841 • Téléc./Fax (613) 562-5338
http://www.uottawa.ca/services/research/rge/index.html
