# Supplementary material for: Provincial prenatal record revision: a multiple case study of evidence-based decision-making at the population-policy level
Source: BMC Health Serv Res. 2008 Dec 19;8:266. doi: 10.1186/1472-6963-8-266 (PMC2642799; doi:10.1186/1472-6963-8-266)
Supplement: Additional file 2 — Ethics approval Laurentian additional file 2. Ethics approval to study from University of Laurentian research ethics board [file 1472-6963-8-266-S2.pdf]

Laurentian University
Université Laurentienne

Research, Development & Creativity Office
Bureau de la recherche, du développement et de la créativité
Tel/Tél.: 705-675-1151, 3944
Fax/Téléc.: 705-671-3850

March 17, 2007

Doctor Phyllis Montgomery,
School of Nursing,
Laurentian University,

This is to inform you that the study entitled *Provincial Prenatal Record Revision: A multiple case study of evidence-based decision-making at the population-policy level* (2007-03-01), presented by Phyllis Montgomery and Nancy Edwards has passed an ethics review by the Laurentian University Research Ethics Board.

Ethics approval is valid until March 17 2008. In March 2008, please submit a request for renewal form to the Office of Research as your your research involving human subjects will continue for longer than one year. Should there be any changes to the project the researcher is required to advise the Laurentian University Research Ethics Board. Please ensure that your research complies with TCPS policies.

A report is due in March 2008 and at the end of the project in December 2008.

Robert Schinke,
Acting President, Laurentian Research Ethics Board,
Department of Human Kinetics,
Laurentian University
