# Supplementary material for: Provincial prenatal record revision: a multiple case study of evidence-based decision-making at the population-policy level
Source: BMC Health Serv Res. 2008 Dec 19;8:266. doi: 10.1186/1472-6963-8-266 (PMC2642799; doi:10.1186/1472-6963-8-266)
Supplement: Additional file 4 — Ethics approval Alberta additional file 4. Ethics approval to study protocol from University of Alberta research ethics board [file 1472-6963-8-266-S4.pdf]

Health Research Ethics Board

213 Heritage Medical Research Centre
University of Alberta, Edmonton, Alberta T6G 2S2
p.780.492.9724 (Biomedical Panel)
p.780.492.0302 (Health Panel)
p.780.492.0459
p.780.492.0839
f.780.492.7808

HEALTH RESEARCH ETHICS APPROVAL FORM

Date: March 2007

Name of Applicant: **Dr. Beverly Williams**

Organization: **U of A**

Department: **Faculty of Nursing**

Project Title: **Provincial prenatal record revision: A multiple case study of evidence-based decision-making at the population-policy level**

The Health Research Ethics Board (HREB) has reviewed the protocol for this project and found it to be acceptable within the limitations of human experimentation. The HREB has also reviewed and approved the subject information letter and consent form.

The approval for the study as presented is valid for one year. It may be extended following completion of the yearly report form. Any proposed changes to the study must be submitted to the Health Research Ethics Board for approval. Written notification must be sent to the HREB when the project is complete or terminated.

Special Comments:

Glenn G. Griener

Dr. Glenn Griener, PhD
Chair of the Health Research Ethics Board
(B: Health Research)

MAR 2 2 2007

Date of Approval Release

File Number: B-320307
