# Supplementary material for: Provincial prenatal record revision: a multiple case study of evidence-based decision-making at the population-policy level
Source: BMC Health Serv Res. 2008 Dec 19;8:266. doi: 10.1186/1472-6963-8-266 (PMC2642799; doi:10.1186/1472-6963-8-266)
Supplement: Additional file 3 — Ethics approval McGill additional file 3. Ethics approval to study protocol from McGill University research ethics board [file 1472-6963-8-266-S3.pdf]

McGill

Faculty of Medicine
3655 Promenade Sir William Osler
Montreal, QC H3G 1Y6

Faculté de médecine
3655, Promenade Sir William Osler
Montréal, QC, H3G 1Y6

Fax/Télécopieur: (514) 398-3595

16 March 2007

Dr. Sonia Semenic
School of Nursing
McGill University
3506 University Street
Montreal, Quebec H3A 2A7

RE: IRB Study Number A03-E09-07B

Dear Dr. Semenic,

Your request for expedited review for the study entitled *Prenatal Record Review: A Multiple Case Study of Evidence-Based Decision-Making at the Population-policy Level,* submitted on behalf of Dr. Nancy Edwards, is complete.

Since this project involves no more than minimal risk, and in accordance with Article 1.6 of the Canadian Tri-Council Policy Statement of Ethical Conduct for Research Involving Humans and U.S. Title 45 CFR 46, Section 110 (b), paragraph (1), we are pleased to inform you that ethics approval for the study protocol (submitted to the IRB on February 27, 2007) is provided on March 16, 2007.

The ethics approval for this project is valid until **March 2008**. The project proposal will be presented for corroborative approval by the Board at the next IRB committee meeting. The Certificate of Ethical Acceptability will be issued to you at that time.

Take note that a review of all research involving human subjects/specimens is required on an annual basis and in conjunction with the date of initial approval. The application for continuing review should be submitted at least one month prior to March 2008. A copy of the continuing review form is available on our website at: http://www.medicine.mcgill.ca/research/irb/forms.htm.

If any modifications occur to the study prior to the scheduled annual review, please inform the IRB promptly.

Sincerely,

Serge Gauthier, MD
Chair
Institutional Review Board

cc: Dr. Nancy Edwards
A03-E09-07B
