# Supplementary material for: Provincial prenatal record revision: a multiple case study of evidence-based decision-making at the population-policy level
Source: BMC Health Serv Res. 2008 Dec 19;8:266. doi: 10.1186/1472-6963-8-266 (PMC2642799; doi:10.1186/1472-6963-8-266)
Supplement: Additional file 5 — Ethics approval Calgary additional file 5. Ethics approval to study protocol from University of Calgary research ethics board [file 1472-6963-8-266-S5.pdf]

FACULTY OF MEDICINE | UNIVERSITY OF CALGARY

OFFICE OF MEDICAL BIOETHICS
Room 93, Heritage Medical Research Bldg
3330 Hospital Drive NW
Calgary, AB, Canada T2N 4N1
Telephone: (403) 220-7990
Fax: (403) 283-8524
Email: omb@ucalgary.ca

2007-03-19

Dr. S.S. Premji
Faculty of Nursing
University of Calgary
PF 2241
Calgary, Alberta

Dear Dr. Premji:

RE: Provincial prenatal record revision: A multiple case study of evidence based decision making at the population-policy level

Ethics ID: E-20799

The above-named research, including the Questionnaire (Key Informant Study Instrument), Consent Form (Version 2.0 Key Informant Information Sheet and Consent Form, Main Contact Information Sheet and Consent Form dated: March 01, 2007), Interview Guide (Semi Structured Interview Guide for Main Contact Persons, Semi Structured Interview Guide for Key Informant), Letters (University of Ottawa Ethics Approval Letter dated February 23, 2007, Recruiting & Permission Letters), Script (Telephone Script: Main Contact Recruitment, Telephone Answering Machine Script: Main Contact Recruitment, Telephone Script: Key Informant Recruitment (given consent), Telephone Script Key Informant Recruitment (publicly available committee list) Telephone Answering Machine Script: Key Informant Recruitment (given consent), Telephone Answering Machine Script: Key Informant Recruitment (publicly available committee list), Email Scripts), and Protocol has been granted ethical approval by the Conjoint Health Research Ethics Board of the Faculties of Medicine, Nursing and Kinesiology, University of Calgary, and the Affiliated Teaching Institutions. The Board conforms to the Tri-Council Guidelines, ICH Guidelines and amendments to regulations of the Food and Drugs Act re clinical trials, including membership and requirements for a quorum.

You and your co-investigators are not members of the CHREB and did not participate in review or voting on this study.
Please note that this approval is subject to the following conditions:
(1) appropriate procedures for consent for access to identified health information have been approved;
(2) a copy of the informed consent form must have been given to each research subject, if required for this study;
(3) a Progress Report must be submitted by **March 19, 2008**, containing the following information:

i) the number of subjects recruited;
ii) a description of any protocol modification;
iii) any unusual and/or severe complications, adverse events or unanticipated problems involving risks to subjects or others, withdrawal of subjects from the research, or complaints about the research;
iv) a summary of any recent literature, finding, or other relevant information, especially information about risks associated with the research;
v) a copy of the current informed consent form;
vi) the expected date of termination of this project.

4) a Final Report must be submitted at the termination of the project.

Please accept the Board's best wishes for success in your research.
Yours sincerely,

Glenys Godlovitch, BA(Hons), LLB, PhD
Chair, Conjoint Health Research Ethics Board

GG/cmcg
c.c. Dr. S. Evans (information) Research Services
Office of Information & Privacy
